# Synchronous bacterial barrier and exudate absorption: A novel dual-function dressing strategy for pin-site infection prevention

**DOI:** 10.1016/j.mtbio.2025.101833

**Published:** 2025-05-08

**Authors:** Bing Liang, Sha Zhou, Linyuan Xue, Qizun Wang, Qianqian Li, Zihan Zheng, Xinyue Ma, Jiyixuan Li, Li Sun, Kunyue Xing, Xiaobo Wen, Xiaolin Wu, Miao Zhang, Dongming Xing

**Affiliations:** a Cancer Institute, The Affiliated Hospital of Qingdao University, Qingdao University, Qingdao, Shandong, 266071, China; b School of Basic Medicine, Qingdao University, Qingdao, Shandong, 266000, China; c School of Pharmacy, Qingdao University, Qingdao, Shandong, 266000, China; d Department of Orthopedics, The Affiliated Hospital of Qingdao University, Qingdao, Shandong, 266003, China; e Department of Ultrasound, The Affiliated Hospital of Qingdao University, Qingdao, Shandong, 266003, China; f Institute of Health Informatics, Faculty of Population Health Sciences, University College London, London, NW1 2DA, United Kingdom; g School of Life Sciences, Tsinghua University, Beijing, 100084, China

**Keywords:** Pin-site infection, Osteomyelitis, *Staphylococcus aureus,* transcriptomic and metabolomic analyses, Exudate absorption

## Abstract

Open pin-site wounds, with infection rates of 11 %–100 %, pose significant clinical challenges, affecting millions globally and often leading to life-threatening complications. Current dressings fail to simultaneously block bacterial invasion and manage internal wound infection, necessitating innovative solutions. This study introduces PINSHIELD, a dual-functional dressing that externally seals wounds while efficiently managing exudate to mitigate pin-site infections (PSI). The external shell provides a physical barrier, while the embedded zinc alginate-polyurethane (ZAPU) layer combines active antibacterial properties with passive bacterial adhesion. The optimized ZAPU structure absorbs exudate and regulates the wound microenvironment, inhibiting bacterial proliferation and limiting infection spread. *In vitro* studies demonstrated that PINSHIELD inhibited *S. aureus* and *E. coli* by 90 %, with a bacterial blocking efficiency exceeding 95 %, significantly outperforming traditional gauze. *In vivo* results showed reduced inflammation, bacterial loads, and *Staphylococcus* abundance, while enhancing microbial diversity and enriching health-associated bacteria. Transcriptomic and metabolomic analyses revealed that PINSHIELD downregulated key *S. aureus* virulence genes (*cna, SSL family, aur*) and disrupted essential metabolic pathways (e.g., fatty acid biosynthesis, aminoacyl-tRNA synthesis), impairing bacterial adhesion, immune evasion, and biofilm formation. By synchronizing bacterial barrier formation with exudate management, PINSHIELD addresses the complex pathological needs of PSI, enhancing therapeutic efficacy and wound healing. This innovative design provides a versatile platform for infection control and personalized wound care, with broad implications for treating open wounds in orthopedic and other invasive device scenarios.

## Highlights

• PINSHIELD integrates a physical pathogen barrier with active antibacterial properties and efficient exudate management, addressing critical clinical challenges in pin-site infection prevention.
• PINSHIELD reshapes the wound microbiome by reducing pathogenic genera and enriching beneficial bacteria, creating a balanced, infection-resistant microenvironment that accelerates wound recovery.
• Through transcriptomic and metabolomic analyses, PINSHIELD downregulates key *S. aureus* virulence genes and disrupts essential metabolic pathways, fundamentally impairing bacterial adhesion, immune evasion, and biofilm formation.
• PINSHIELD synchronizes infection control and wound healing, offering a versatile orthopedic/invasive-device wound management platform integrating materials science with precision care.

## Graphical abstract

**Figure:**
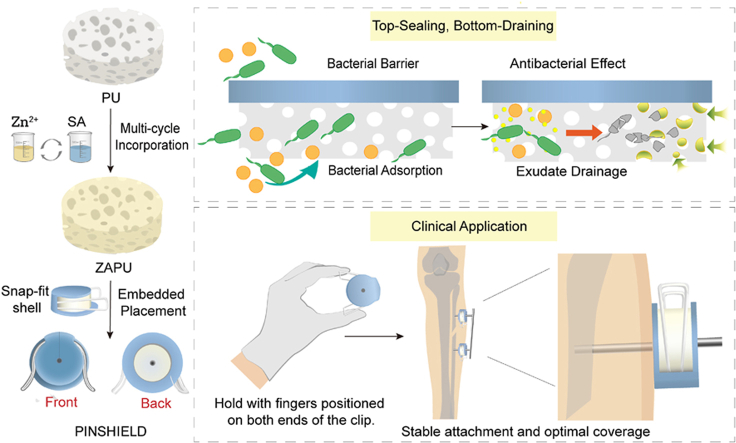

## 1 Introduction

Pin-site wounds, characterized by their open nature and deep penetration into the medullary cavity, pose a significant challenge in orthopedics [1]. With over 186 million fractures occurring annually worldwide [2], many patients require external fixation devices, resulting in millions of open pin-site wounds each year [3,4]. These wounds are highly susceptible to bacterial invasion, particularly *Staphylococcus aureus* (*S.aureus*), which forms biofilms that exacerbate infections and trigger severe local and systemic inflammatory responses [1,[4], [5], [6], [7], [8]]. The incidence of pin-site infection (PSI) ranges from 11 % to 100 % [4,9,10], often leading to complications such as osteomyelitis, pin loosening, and fixation failure [[11], [12], [13], [14]], significantly prolonging healing time, increasing hospitalization, and raising medical costs [4,15]. Given the widespread clinical prevalence of PSI, there is an urgent need for innovative therapeutic strategies to effectively manage these infections.

Currently, common wound care methods, such as gauze dressings, while effective at covering the wound, fail to simultaneously block bacterial invasion and manage inflammatory exudates, thus hindering infection control and wound healing [[16], [17], [18], [19]]. Furthermore, existing technologies typically lack the ability to regulate wound microbiomes, making it difficult to balance antibacterial efficacy with tissue repair needs [20]. Therefore, the development of personalized dressings that can provide effective bacterial barriers and efficiently manage wound exudate is a critical requirement for controlling PSI [21].

To address these challenges, we propose a novel dual-functional dressing strategy—PINSHIELD—designed to meet the complex pathological demands of pin-site wounds. PINSHIELD integrates two core functions: “Top-Sealing” and “Bottom-Draining.” The “Top-Sealing” function uses an external clip-fit shell to create a sealed environment, blocking pathogen invasion. Simultaneously, the embedded zinc alginate-polyurethane (ZAPU) porous material offers both active antibacterial properties and passive bacterial capture, providing dual protection for the wound. The “Bottom-Draining” function optimizes the ZAPU structure to efficiently absorb exudate, regulate the moist microenvironment, inhibit bacterial proliferation, and reduce infection risk. These synchronized functions enable PINSHIELD to address the high infection risks and open nature of pin-site wounds while balancing external protection with internal infection management. PINSHIELD consists of two components: an external elastic medical rubber shell and embedded ZAPU antimicrobial material. The shell provides mechanical protection and a closed barrier, while the ZAPU layer captures and eliminates bacteria, absorbs exudates, and maintains a moist wound environment. This design ensures an optimal balance between efficient antibacterial effects and wound moisture regulation, significantly improving the management of pin-site wounds.

We validated the multifunctional performance of PINSHIELD through *in vitro* and in vivo experiments. *In vitro* tests demonstrated that PINSHIELD achieved a 90 % inhibition rate against *S.aureus* and *Escherichia coli* (*E.coli*), with bacterial blocking efficiency exceeding 95 %, while significantly outperforming traditional gauze in exudate absorption. *In vivo* studies showed that PINSHIELD notably reduced bacterial loads around the pin-site, decreased *Staphylococcus* abundance, and enhanced the diversity and richness of health-associated bacteria, such as *Actinomyces* and *Porphyromonas*. Transcriptomic and metabolomic analyses further revealed PINSHIELD's antimicrobial mechanism: by downregulating key virulence genes of *S.aureus* (e.g., *cna,* SSL family*, aur*) and associated metabolites (e.g., styrene, serotonin), PINSHIELD inhibits critical metabolic pathways, including fatty acid biosynthesis, antibiotic synthesis, aminoacyl-tRNA synthesis, bacterial secretion systems, phosphotransferase systems (PTS), and amino acid metabolism. These effects disrupt bacterial virulence, immune evasion mechanisms, and biofilm formation, ultimately blocking bacterial growth and preventing the progression of infection.

In conclusion, PINSHIELD's innovative design and multifunctionality offer a breakthrough solution for PSI management. Its simple and practical design not only holds significant clinical application potential but also offers a new approach to managing other open wounds, such as those caused by drainage tubes and catheters.

## 2 Results

### 2.1 PINSHIELD fabrication and characterization

#### 2.1.1 PINSHIELD design strategy

**Fig. 1:**
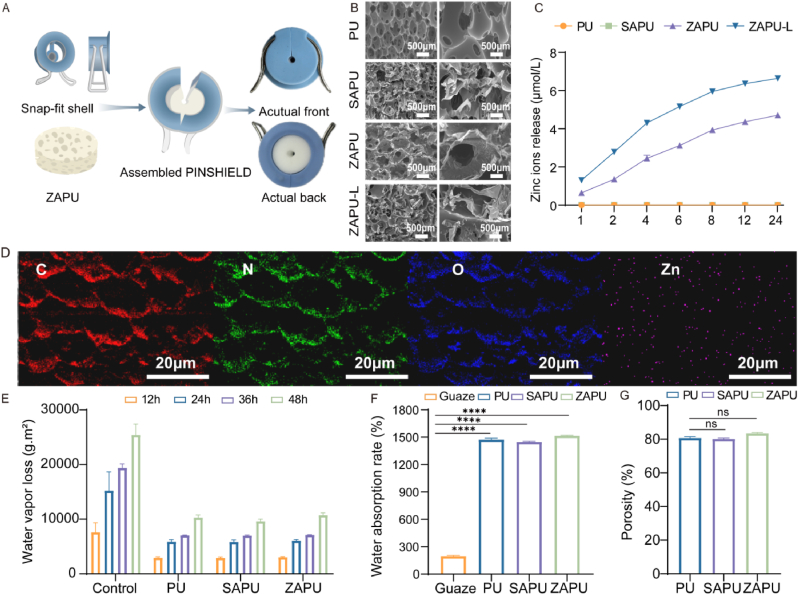
**Design and Characterization of PINSHIELD and ZAPU.** A) Schematic illustration and physical image of PINSHIELD assembly. B) SEM images of PU, SAPU, ZAPU, and ZAPU-L at 50X and 200× magnifications. C) Zinc ion release profile of ZAPU. D) Elemental mapping of ZAPU. E) Water vapor transmission loss of PU, SAPU, and ZAPU at 12, 24, 36, and 48 h. F) Water absorption rate of Gauze, PU, SAPU, and ZAPU. G) Porosity of PU, SAPU, and ZAPU (∗∗∗∗p < 0.0001).

PINSHIELD consists of two components: an external shell and an internal antimicrobial material (Fig. 1A). The external shell is designed to securely fit the wound, adhering to the skin surface with medical adhesive. The total weight of PINSHIELD is 15.2 ± 0.5 g. The core functions of “Top-Sealing” and “Bottom-Draining” are realized via the clip-fit shell, ensuring rapid and stable wound coverage, which enhances the dressing's ease of application while providing effective sealing and protection. The design prioritizes patient comfort, allowing for painless removal and easy replacement, which significantly reduces infection risks and patient discomfort. The internal structure integrates both active and passive antibacterial mechanisms, forming a sealed barrier that effectively prevents the intrusion of exogenous pathogens.

#### 2.1.2 Zinc alginate polyurethane (ZAPU) preparation and characterization

The internal functional layer of PINSHIELD consists of a ZAPU sponge, made by coating a medical polyurethane (PU) sponge base with antimicrobial materials. The preparation involves alternately immersing the PU sponge in zinc ion and alginate solutions for short cross-linking periods, followed by freeze-drying. The alginate gel's adhesive properties and the antimicrobial action of zinc ions provide the ZAPU dressing with enhanced bacterial capture and eradication capabilities.

Fig. 1B presents SEM images of PU and its modified variants: SAPU (PU modified with sodium alginate), ZAPU-L (PU modified with sodium alginate and zinc ions via a blended cross-linking method), and ZAPU (PU modified with sodium alginate and zinc ions via a sequential cross-linking method). Both PU and ZAPU exhibit a porous and interconnected structure. Moreover, ZAPU retains excellent porosity after modification, with the surface displaying a gel-like, wrinkled morphology that indicates the zinc alginate gel layer adheres to the sponge surface post-freeze-drying, resulting in an uneven interface. Compared with the blended cross-linked ZAPU-L, ZAPU displays a more stable and well-defined porous structure. To further investigate the performance of these materials under physiologically relevant conditions, SEM analysis was also conducted after porcine serum absorption and controlled drying (Fig. S1A). PU, SAPU, and ZAPU-L exhibited significant structural degradation, with collapsed pores and surface fragmentation, while ZAPU largely preserved its original porous morphology, demonstrating its superior structural resilience in exudate-rich environments.

Fig. 1C illustrates the zinc ion release profile of ZAPU. As immersion time increases, zinc ion release also increases, indicating that ZAPU can provide a sustained, gradual release of zinc ions, enhancing its antimicrobial effects. This sustained release is more consistent than that of the gel-modified ZAPU-L. Fig. 1D presents transmission electron microscopy (TEM) and energy-dispersive X-ray (EDX) mapping images, confirming the successful incorporation of zinc ions into the PU matrix. Fig. 1E shows the water vapor transmission rate (WVTR) of ZAPU at 2875.4 g m^2^/day, significantly lower than the blank group (14708.9 g m^2^/day), reducing moisture loss by 80.45 % and bringing it within the ideal range of 2000–2500 g m^2^/day. Compared to PU and SAPU, ZAPU maintains an ideal WVTR, which helps regulate wound moisture and prevents excessive dehydration.

Fig. 1F displays the liquid absorption rates of different materials. Gauze absorbs 1.2 times its dry weight, whereas ZAPU absorbs 15 times its dry weight, significantly outperforming gauze and demonstrating superior absorption capacity. Serum absorption tests under clinically simulated conditions (Fig. S1B) revealed that ZAPU absorbed porcine serum at levels significantly exceeding gauze, with efficacy consistent with its water retention performance. This enhanced serum uptake underscores ZAPU's superior exudate management capacity compared to conventional materials. Porosity plays a key role in biomaterial performance, impacting liquid absorption, moisture retention, and vapor permeability. ZAPU has a porosity of 83.14 ± 0.3 %, higher than PU (80.96 ± 1.3 %) and SAPU (80.10 ± 0.5 %) (Fig. 1G), indicating that the short multi-layer modification process enhances pore connectivity, increasing the sponge's surface area and improving ethanol absorption. SEM analysis confirms ZAPU's regular, intact porous structure, highlighting its efficiency in absorbing exudates for wound care applications.

“Bottom-Draining” are realized via the clip-fit shell, ensuring rapid and stable wound coverage, which enhances the dressing's ease of application while providing effective sealing and protection. The design prioritizes patient comfort, allowing for painless removal and easy replacement, which significantly reduces infection risks and patient discomfort. The internal structure integrates both active and passive antibacterial mechanisms, forming a sealed barrier that effectively prevents the intrusion of exogenous pathogens.

### 2.2 *In vitro* evaluation of PINSHIELD's synergistic antibacterial properties and biocompatibility

**Fig. 2:**
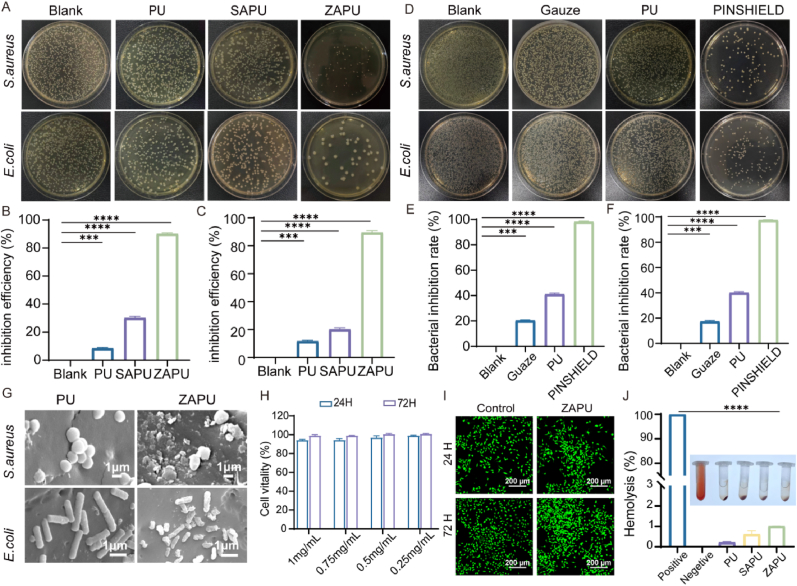
**Antibacterial Efficacy and Biocompatibility Evaluation of PINSHIELD.** A) Colony count of *S.aureus* and *E.coli* on PU, SAPU, and ZAPU dressings. B) Antibacterial rate on *S.aureus* (ZAPU vs Blank, ∗∗∗p < 0.001, ∗∗∗∗p < 0.0001). C) Antibacterial rate on *E.coli* (ZAPU vs Blank, ∗∗∗p < 0.001, ∗∗∗∗p < 0.0001). D) Colony count of unblocked *S.aureus* and *E.coli* on Gauze, PU, and PINSHIELD. E) Inhibition rate of *S.aureus* (PINSHIELD vs Blank, ∗∗∗p < 0.001, ∗∗∗∗p < 0.0001). F) Inhibition rate of *E.coli* (PINSHIELD vs Blank, ∗∗∗p < 0.001, ∗∗∗∗p < 0.0001). G) Representative SEM images (scale bar = 1 μm) showing the morphology and surface adherence of *S. aureus* and *E. coli* after 2-h co-culture with PU and ZAPU. H) Cell viability of L929 cells after treatment with ZAPU. I) Live/dead cell staining results of L929 cells treated with ZAPU at 24h and 72h. J) Hemolysis evaluation of PU, SAPU, and ZAPU (ZAPU vs Positive control, ∗∗∗∗p < 0.0001).

The skin serves as the body's first line of defense, preventing the entry of harmful microorganisms. However, during external fixation surgery for fractures, this natural barrier is compromised, making the wound vulnerable to bacterial infection. In this study, *S.aureus* and *E*.*coli* were selected as typical pathogens associated with PSI to assess the antibacterial efficacy of the dressing. Fig. 2A shows bacterial colony counts, demonstrating ZAPU's active bactericidal properties. Compared to the blank control, PU and SAPU exhibited inhibition rates of 8.5 % ± 1.0 and 30.2 % ± 2.0 against *S.aureus*, and 11.25 % ± 0.9 and 36.36 % ± 1.9 against *E.coli*, respectively (Fig. 2B and C). ZAPU exhibited significantly superior antibacterial performance, with inhibition rates of 90.23 % ± 0.5 against *S.aureus* and 91.23 % ± 1.2 against *E.coli* (Fig. 2B and C), primarily attributed to zinc ions' suppression of bacterial biofilm formation and metabolic interference. To further simulate infection risk following exudate absorption, the standard colony-forming unit (CFU) counting method was also conducted using serum-soaked materials. ZAPU exhibited markedly higher antibacterial activity against both *S. aureus* and *E. coli* immediately after serum absorption compared to PU and SAPU. Specifically, ZAPU showed an inhibition rate of 83.3 % against *S. aureus* and 78.4 % against *E. coli*, while SAPU demonstrated 64.2 % inhibition against *S. aureus* and 57.6 % against *E. coli*. PU showed lower inhibition rates of 32.66 % against *S. aureus* and 30.23 % against *E. coli.* These results reinforce ZAPU's superior antibacterial efficacy, even under biomimetic conditions.

For passive antibacterial properties, a bacterial capture experiment using a hyaluronic acid filter membrane (HAFM) assessed bacterial-blocking efficiency (Fig. 2D). PINSHIELD achieved 99 % bacterial blocking efficiency for both *S.aureus* and *E.coli*, significantly outperforming traditional gauze (Fig. 2E and F). The physical barrier of PINSHIELD effectively blocked bacterial invasion; however, in short-term tests, antimicrobial factors from the inner layer did not fully exert their bactericidal effects. In contrast, gauze showed only 20 % ± 0.4 inhibition against *S.aureus* and 16.8 % ± 1.6 against *E.coli* (Fig. 2E and F).

To assess bacterial adherence and in situ antibacterial behavior, PU and ZAPU were co-cultured with bacterial suspensions and observed using SEM. SEM analysis demonstrated marked antibacterial effects of ZAPU compared to unmodified PU (Fig. 2G). *S. aureus* on ZAPU surfaces showed collapsed spherical morphology with extensive cell wall rupture, cytoplasmic leakage, and severe deformation, while *E. coli* exhibited structural disintegration characterized by twisted cellular bodies and irregular fragmentation. In contrast, both bacterial species maintained intact morphology on PU surfaces: *S. aureus* retained smooth, tightly clustered spherical structures, and *E. coli* preserved characteristic rod-shaped configurations with undamaged cell walls. These findings confirm ZAPU's ability to disrupt bacterial membrane integrity, whereas PU showed negligible bactericidal activity under identical conditions.

To further assess biocompatibility, L929 cell viability was evaluated using the zinc alginate gel coating (Fig. 2H). Results showed that ZAPU extracts did not significantly affect cell viability, confirming its excellent biocompatibility. Additionally, live/dead staining and hemolysis tests confirmed that PINSHIELD exhibited no cytotoxicity, with a hemolysis rate of only 1 % ± 0.1 %, well below clinical safety standards (Fig. 2I and J).

### 2.3 PINSHIELD's impact on tissue protection and systemic inflammatory response in pin-site wounds

**Fig. 3:**
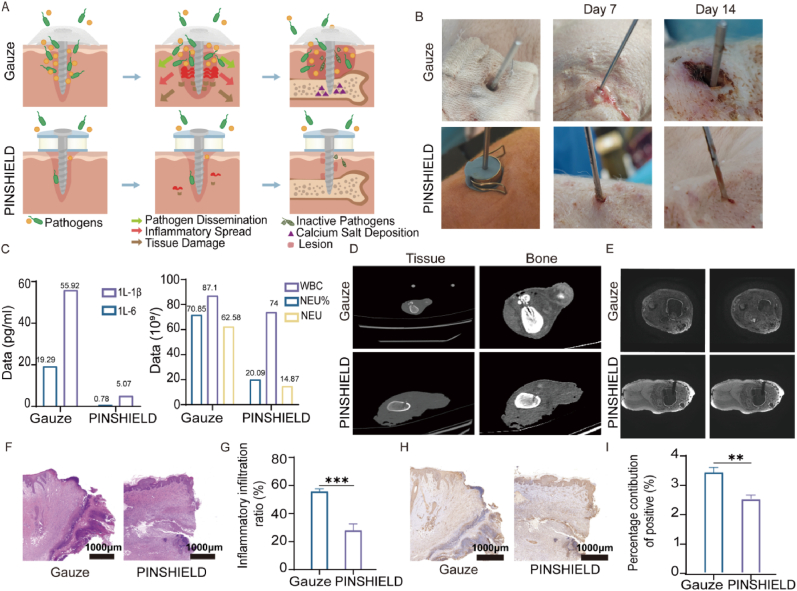
**Infection and Inflammation Control in Pin-Site Wounds by PINSHIELD.** A) Schematic representation of pin-site infection (PSI) progression and treatment strategies with PINSHIELD and gauze. B) Gross observations of pin-site wounds on Day 7 and Day 14. C) Peripheral blood was collected from the ear vein of animals on Day 7 for white blood cell (WBC) and neutrophil (NEU) counts, as well as for the quantification of inflammatory cytokines (IL-6 and IL-1β). D) CT imaging showing pin-site and bone structure conditions on Day 14. E) MRI using T2-weighted sequences depicting soft tissue and medullary cavity conditions on Day 14. F) Histological analysis using HE staining to assess inflammatory cell infiltration and tissue necrosis (scale bar = 1000 μm). G) Semi-quantitative analysis of inflammatory infiltration analyzed by HE staining (∗∗∗p < 0.001). H) IHC staining for IL-1β to evaluate inflammatory response in pin-site tissues (scale bar = 1000 μm). I) Semi-quantitative analysis of IHC staining data presented as bar charts (∗∗p < 0.01).

To compare infection control efficacy between PINSHIELD and conventional gauze, we created a schematic of infection progression, highlighting PINSHIELD's inhibitory effects on PSI (Fig. 3A). On Day 0, after bone pin implantation and inoculation with *S.aureus*, wounds were treated with either gauze or PINSHIELD (Fig. 3B). On Day 7, the gauze group exhibited significant wound enlargement, erythema, edema, and yellow purulent exudates, indicating severe infection and potential deep tissue involvement (Fig. 3B). In contrast, PINSHIELD-treated wounds appeared clean with mild erythema and no exudates, reflecting effective infection control. By Day 14, infection in the gauze group worsened, showing intensified erythema, tissue congestion, necrosis, and purulent discharge, consistent with osteomyelitis. In contrast, PINSHIELD-treated wounds showed minimal redness, no abnormal discharge, and reduced infection (Fig. 3B).

Inflammatory markers were significantly elevated in the gauze group, with white blood cell (WBC) counts at 71.85 × 10^9^/L, neutrophil percentage (GRA%) at 87.1 %, and IL-6/IL-1β levels at 55.92 pg/mL and 19.29 pg/mL, respectively. In contrast, the PINSHIELD group showed lower levels, with WBC at 20.09 × 10^9^/L (19.41 ± 6.53), GRA% at 74 %, and reduced IL-6 (5.07 pg/mL) and IL-1β (0.78 pg/mL) levels on Day 7 (Fig. 3C). Imaging studies on Day 14 revealed significant differences in infection control between the groups. CT scans of the gauze group showed high-density inflammatory barriers at the pin sites, indicating calcified deposits, with low-density areas suggesting exudate accumulation and abscess formation, confirming osteomyelitis (Fig. 3D). Additionally, low-density signals in the bone marrow and pin loosening suggested infection spread to deeper tissues, compromising bone stability. MRI scans of the gauze group showed high-signal intensity at the pin sites, indicative of soft tissue infection extending from the medullary cavity, with patchy high-signal areas representing residual infection. Inflammatory pseudomembranes and low-signal regions suggested gas gangrene. In contrast, PINSHIELD-treated sites showed no abnormalities in CT or MRI, with clean pin sites, uniform bone marrow density, and intact cortical bone (Fig. 3D and E). Histological assessments further confirmed the results. HE staining in the gauze group revealed significant inflammation, including extensive cell infiltration and necrosis (Fig. 3F), while PINSHIELD-treated tissues exhibited minimal inflammatory infiltration, indicating effective infection control. IL-1β immunohistochemistry corroborated these findings, with significantly lower IL-1β expression in PINSHIELD-treated tissues (Fig. 3G), reflecting reduced inflammation.

### 2.4 Regulation of microbial load and diversity in pin-site wounds by PINSHIELD

**Fig. 4:**
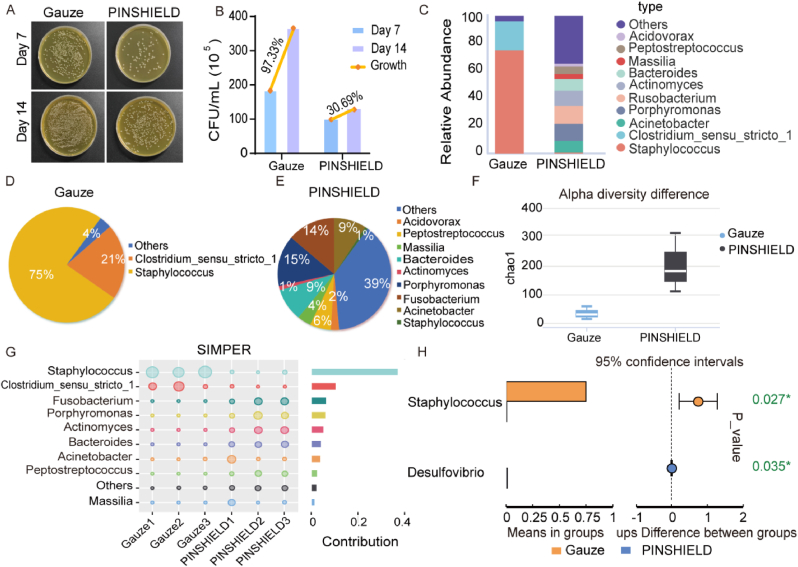
**Microbial Community Modulation by PINSHIELD in Pin-Site Wounds.** A) Representative images of bacterial colonies on agar plates from pin-site wound samples collected on Days 7 and 14. B) Quantification of microbial load at pin sites using CFU counts, comparing bacterial growth between PINSHIELD and gauze groups on Days 7 and 14. C) Taxonomic composition of microbial communities at the genus level based on 16S rRNA sequencing from pin-site wounds on Day 14, comparing PINSHIELD and gauze groups. D) Pie chart showing genus-level microbial composition in the gauze group, highlighting the predominance of pathogenic genera. E) Pie chart showing genus-level microbial composition in the PINSHIELD group, illustrating an increase in beneficial genera and a reduction in pathogenic bacteria. F) Alpha diversity analysis comparing microbial diversity indices between PINSHIELD and gauze groups. G) Simper analysis highlighting changes in the relative abundance of pathogens and infection-resistant genera. H) T-test analysis showing increased levels of repair-associated genera, such as *Desulfovibrio*, in PINSHIELD-treated wounds.

On Days 7 and 14, microbial load in pin-site wounds was significantly reduced in the PINSHIELD group compared to the gauze group, confirming PINSHIELD's ability to inhibit bacterial proliferation and prevent infection progression (Fig. 4A and B). Colony-forming unit (CFU) counts indicated an increase in bacterial load in the gauze group, while bacterial levels remained stable in the PINSHIELD group, highlighting its sustained inhibitory effect (Fig. 4B).

16S rRNA sequencing revealed that PINSHIELD reduced pathogenic bacteria load while enhancing the diversity of beneficial microbiota (Fig. 4C–E). Notably, pathogenic genera such as *Staphylococcus* and *Clostridium*were were less abundant in the PINSHIELD group, while genera associated with health, such as *Actinomyces* and *Porphyromonas*, were significantly enriched (Fig. 4D and E). These results underscore PINSHIELD's potential to rebalance the microbial community, fostering a healthier wound environment. Alpha diversity analysis demonstrated significantly higher diversity in the PINSHIELD group, suggesting a more robust microbial composition (Fig. 4F). Simper analysis revealed that PINSHIELD reduced *Staphylococcus* abundance while enriching infection-resistant genera like *Porphyromonas* and *Fusobacterium* (Fig. 4G), contributing to microbial stability. Further T-test analysis confirmed PINSHIELD's role in increasing the abundance of repair-associated genera, such as *Desulfovibrio*, reinforcing its impact on pathogen control and microbial balance (Fig. 4H). Together, these results demonstrate PINSHIELD's ability to promote an anti-infective microenvironment by reshaping the wound microbiome.

### 2.5 Inhibition of *S.aureus* virulence factors and biofilm formation by PINSHIELD

**Fig. 5:**
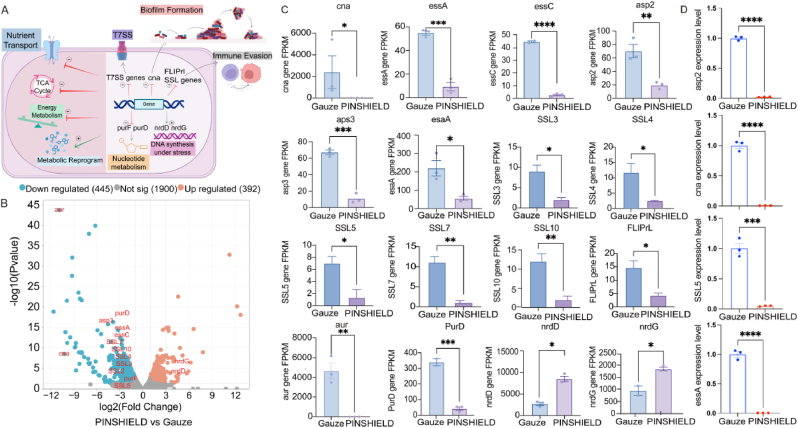
**Gene Expression Modulation and Virulence Suppression in S.aureus by PINSHIELD.** A) Schematic illustrating PINSHIELD-induced gene expression alterations in *S.aureus*. B) Volcano plot showing DEGs in the PINSHIELD group compared to the gauze group, with significantly upregulated and downregulated genes highlighted. C) Expression levels of key DEGs associated with virulence, biofilm formation, immune evasion, and metabolic activity. Bars indicate relative expression levels, with significance denoted by stars (∗p < 0.05, ∗∗p < 0.01, ∗∗∗p < 0.001, ∗∗∗∗p < 0.0001). D) qPCR validation of selective RNA-seq-identified DEGs (*Cna, Asp2, essA, SSL5*) in *S. aureus* from pin-site wounds. Results confirmed consistent expression trends with RNA-seq data. Expression levels were normalized to the internal reference gene 16S rRNA, and relative expression was calculated using the 2^–ΔΔCt method. Data are presented as mean ± SD (n = 3) (∗∗∗p < 0.001, ∗∗∗∗p < 0.0001).

To evaluate the inhibitory effects of PINSHIELD on the pathogenicity of *S.aureus*, transcriptomic analysis was performed on bacteria isolated from pin sites. A schematic illustrating PINSHIELD-induced gene expression alterations and metabolic reprogramming in *S.aureus* is shown in Fig. 5A. PINSHIELD treatment significantly downregulated 445 genes and upregulated 392 genes compared to the gauze group (Fig. 5B). Key virulence genes associated with biofilm formation, such as *cna* (encoding collagen-binding adhesin), were markedly downregulated, indicating PINSHIELD's role in reducing bacterial adhesion and biofilm formation (Fig. 5B–C,D). Genes integral to the Type VII Secretion System (T7SS), including *essA, essC*, *asp2*, *asp3*, and *esaA*, were also significantly downregulated, further supporting PINSHIELD's efficacy in impairing biofilm development and stability (Fig. 5B–C, D). PINSHIELD also suppressed genes involved in immune evasion. SSL family genes (*SSL3*, *SSL4*, *SSL5*, *SSL7*, and *SSL10*), which encode superantigen-like toxins that neutralize host immune factors, were substantially downregulated, highlighting PINSHIELD's impact on diminishing bacterial immune evasion (Fig. 5B–C, D). Additionally, the downregulation of *aur* (encoding aureolysin) and *FLIPrl* (inhibiting phagocytosis by host) further indicated enhanced immune clearance and reduced secretion of virulence factors (Fig. 5B and C). The downregulation of *purD*, involved in purine biosynthesis, indicates that PINSHIELD disrupts nucleotide metabolism, impairing bacterial proliferation (Fig. 5B and C). However, the upregulation of *nrdD* and *nrdG* suggests a compensatory response aimed at maintaining DNA synthesis and bacterial viability (Fig. 5B and C).

**Fig. 6:**
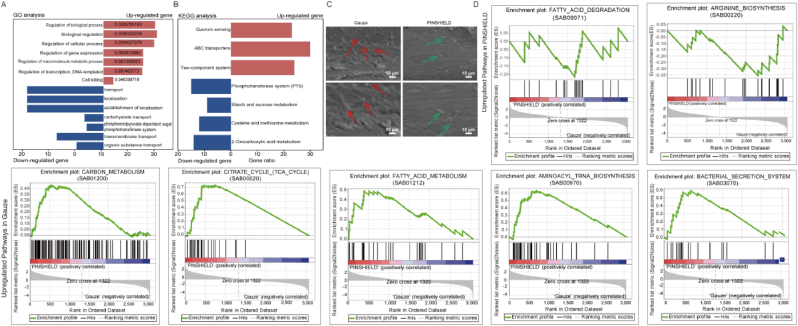
**Functional Analysis of DEGs and Biofilm Suppression by PINSHIELD.** A) GO enrichment analysis of DEGs identified in the PINSHIELD group, highlighting upregulated and downregulated categories. B) KEGG pathway analysis of DEGs, showing significant downregulation and upregulation of pathways. C) SEM images of bone pin surfaces from gauze- and PINSHIELD-treated groups, illustrating extensive biofilm formation and extracellular polymeric substances (EPS) in the gauze group (red arrows) and minimal biofilm formation with scattered bacterial cells in the PINSHIELD group (green arrows). D) GSEA of DEGs, illustrating pathway enrichment differences between the PINSHIELD and gauze groups. (For interpretation of the references to colour in this figure legend, the reader is referred to the Web version of this article.)

Gene Ontology (GO) analysis revealed that upregulated genes were primarily associated with regulation of biological and cellular processes (Fig. 6A). Downregulated genes were enriched in nutrient uptake and energy metabolism pathways, indicating PINSHIELD's role in curbing bacterial growth by inhibiting nutrient acquisition (Fig. 6A). Kyoto Encyclopedia of Genes and Genomes (KEGG) pathway analysis revealed significant downregulation of energy metabolism pathways, such as the phosphotransferase system (PTS) and 2-oxocarboxylic acid metabolism, undermining the survival of *S.aureus* (Fig. 6B). Conversely, upregulated pathways related to quorum sensing and ABC transporters indicated a bacterial adaptive response, though these mechanisms failed to overcome PINSHIELD's antimicrobial effects (Fig. 6B).

Gene Set Enrichment Analysis (GSEA) revealed that PINSHIELD significantly suppressed metabolic and virulence pathways in *S.aureus*. In the gauze group, upregulated genes were linked to critical metabolic pathways such as carbon metabolism, TCA cycle, and fatty acid metabolism, promoting biofilm formation and bacterial proliferation (Fig. 6D). In contrast, PINSHIELD treatment upregulated stress-related pathways like fatty acid degradation and arginine biosynthesis, suggesting bacterial attempts to survive under metabolic stress (Fig. 6D). However, these compensatory mechanisms were insufficient to counteract PINSHIELD's broad-spectrum antimicrobial effects.

SEM analysis revealed marked differences in biofilm formation. The gauze group exhibited extensive biofilm formation with well-developed three-dimensional structures and extracellular polymeric substances (EPS) (Fig. 6C, red arrows). In contrast, PINSHIELD showed minimal biofilm formation, with isolated bacterial cells, demonstrating its potent effect on bacterial adhesion and biofilm inhibition (Fig. 6C, green arrows).

### 2.6 Multi-dimensional metabolic suppression of *S.aureus* by PINSHIELD

**Fig. 7:**
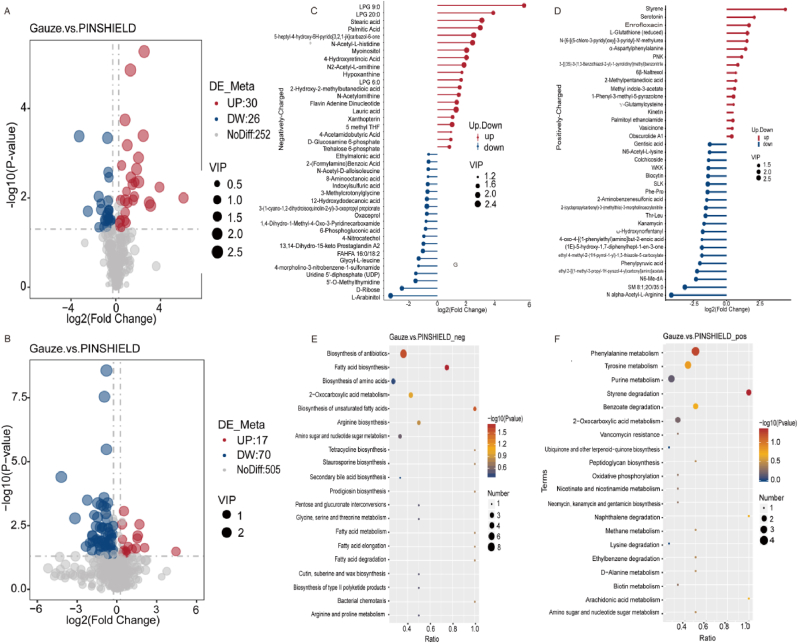
**Metabolomic Profiling of Differentially Expressed Metabolites in PINSHIELD-Treated *S.aureus****.* A) Volcano plot of negatively-charged metabolites between gauze and PINSHIELD groups. B) Volcano plot of positively-charged metabolites between gauze and PINSHIELD groups. C) Analysis of key negatively-charged metabolites, highlighting reductions in biofilm-associated lipids and increases in energy-related metabolites in the PINSHIELD group. D) Analysis of key positively-charged metabolites, showing adaptive metabolic responses in the PINSHIELD group. E) KEGG pathway enrichment analysis of negatively-charged metabolites, highlighting downregulation of pathways including fatty acid biosynthesis, antibiotics biosynthesis, and 2-oxocarboxylic acid metabolism in the PINSHIELD group. F) KEGG pathway enrichment analysis of positively-charged metabolites, showing inhibition of pathways such as phenylalanine metabolism, tyrosine metabolism, styrene degradation, and benzoate degradation in the PINSHIELD group.

Metabolomic analysis revealed significant changes in the metabolic profiles of *S*.*aureus* in PINSHIELD-treated versus gauze groups (Fig. 7A and B). Increased levels of lipid and fatty acid metabolites such as long-chain lysophosphatidylglycerols (LPG 9:0, LPG 20:0), stearic acid, and palmitic acid, associated with virulence and biofilm formation were observed in the gauze group, indicating *S.aureus*’ use of active lipid metabolism to enhance biofilm formation (Fig. 7C). Elevated positively-charged metabolites like styrene and serotonin suggested oxidative stress and potential antibiotic resistance (Fig. 7D).

In contrast, the PINSHIELD-treated group showed upregulation of metabolites associated with energy and nucleotide metabolism, such as L-arabinitol, D-ribose, and UDP, providing limited energy reserves under antibacterial pressure (Fig. 7C). Increased levels of N-alpha-acetyl-L-arginine and sphingomyelin reflected adaptive regulation of amino acid and lipid metabolism (Fig. 7D). Metabolic reprogramming, as evidenced by the increase in phenylpyruvic acid and other metabolites, further underscores PINSHIELD's efficacy in disrupting core metabolic pathways to restrict bacterial growth and virulence expression.

**Fig. 8:**
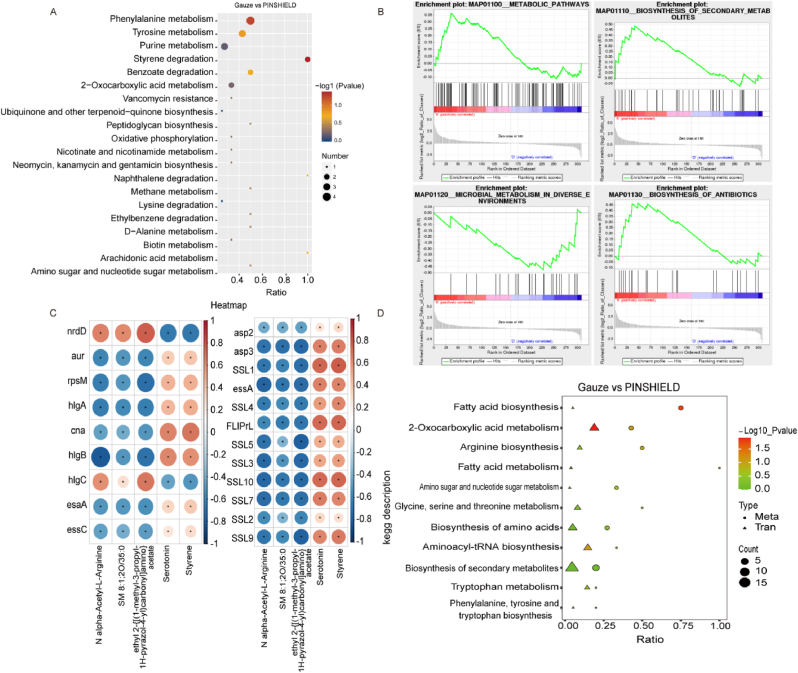
**Integrated Transcriptome and Metabolome Analysis of PINSHIELD's Effects on *S.aureus****.* A) GSEA enrichment analysis of differentially expressed metabolites, highlighting downregulated pathways in the PINSHIELD group compared to the gauze group. B) Focused GSEA analysis of key suppressed pathways, emphasizing PINSHIELD's extensive metabolic regulation. C) Transcriptome-metabolome correlation analysis showing associations between key DEGs and deferentially expressed metabolites, revealing PINSHIELD's regulatory effects on metabolic activity. D) Combined KEGG and GSEA enrichment analyses demonstrating comprehensive inhibition of critical biosynthetic and metabolic pathways by PINSHIELD.

KEGG pathway enrichment analysis corroborated these findings, demonstrating significant downregulation of key metabolic pathways in the PINSHIELD group, including biosynthesis of antibiotics, fatty acid biosynthesis, unsaturated fatty acid biosynthesis, and 2-oxocarboxylic acid metabolism (Fig. 7E), impairing bacterial membrane synthesis and biofilm stability. Additionally, phenylalanine and tyrosine metabolism were inhibited, indicating PINSHIELD's disruption of DNA synthesis and metabolic homeostasis, further curbing bacterial adaptation (Fig. 7F). Differential metabolite GSEA enrichment analysis confirmed extensive metabolic suppression by PINSHIELD, with significant downregulation across metabolic pathways, secondary metabolite biosynthesis, and microbial metabolism (Fig. 8A and B). These findings align closely with KEGG pathway results (Fig. 7E and F), highlighting PINSHIELD's comprehensive inhibition of *S.aureus* metabolism, particularly in pathways associated with virulence and biofilm formation.

Transcriptome-metabolome integrated analysis revealed significant correlations between key genes and specific metabolites, providing deeper insights into PINSHIELD's regulatory effects on *S.aureus* metabolic activity in PSI. Upregulation of *nrdD* indicated a bacterial compensatory response under stress, while downregulation of virulence-related genes such as *cna* and SSL family genes, along with negative correlations with metabolites like styrene and serotonin, underlined PINSHIELD's ability to weaken bacterial adhesion and immune evasion (Fig. 8C). The downregulation of *aur* and its negative correlation with N-alpha-acetyl-L-arginine further reinforced PINSHIELD's ability to disrupt bacterial virulence through metabolic adjustments (Fig. 8C).

KEGG and GSEA analyses of transcriptomic and metabolomic data provided evidence of PINSHIELD's pathway inhibition, including fatty acid biosynthesis, amino acid synthesis, aminoacyl-tRNA biosynthesis, and secondary metabolite synthesis (Fig. 8D). These disruptions compromised bacterial membrane integrity, protein synthesis, and environmental adaptability, illustrating PINSHIELD's broad-spectrum antimicrobial effects and potential in preventing PSI and controlling bacterial virulence.

## 3 Discussion

This study introduces PINSHIELD, a novel personalized dressing for pin-site wounds, and validates its multifaceted efficacy in inhibiting *S*.*aureus* metabolic activity and virulence factors. Our findings highlight PINSHIELD's significant advantages in physical barrier function, antibacterial activity, exudate management, microbiome modulation, and suppression of critical metabolic and molecular pathways, positioning it as an innovative solution for comprehensive infection control in pin-site wounds.

The core innovation of PINSHIELD lies in its multi-mechanistic synergy, combining both physical and biochemical suppression to establish a comprehensive anti-infection defense. The outer clip-fit shell of PINSHIELD functions as an effective physical barrier, preventing external pathogens from entering the wound, while the ZAPU's porous structure captures and traps bacteria. PINSHIELD demonstrated significantly better anti-permeation and bacterial trapping performance compared to traditional gauze dressings [22,23]. The embedded ZAPU layer further enhances its antibacterial activity by releasing zinc ions that disrupt bacterial membrane integrity and interfere with metabolic processes, effectively inhibiting pathogen proliferation [24]. CFU counts confirmed that ZAPU exhibited superior antibacterial activity against both *S.aureus* and *E.coli* compared to PU and SAPU, while PINSHIELD as a whole outperformed both gauze and PU dressings, demonstrating its excellent bacterial blocking efficiency [25,26].

Exudate management is essential for controlling infection in pin-site wounds, and PINSHIELD excels in this regard [27,28]. The ZAPU sponge, with its hydrophilic surface and porous structure, efficiently absorbs exudate, creating a dry wound environment that prevents bacterial proliferation. This dual action not only reduces infection risk but also supports wound healing, as further evidenced by the significant reduction in local inflammatory markers, such as IL-1β, observed in our in vivo studies [[29], [30], [31]]. Together, these features demonstrate that the physical barrier and active antibacterial properties of PINSHIELD work synergistically to prevent infection escalation and promote healing.

This study provides the first systematic investigation of microbial community shifts within pin-site wounds [32]. Using 16S rRNA sequencing, we demonstrated that PINSHIELD modulates the microbiome by reducing pathogenic bacteria, such as *Staphylococcus*, and enhancing beneficial genera like *Actinomyces*. The reshaping of the microbial community in PINSHIELD-treated wounds supports the hypothesis that enhanced microbial diversity provides a protective barrier against infection, fostering more effective wound recovery and reducing complications like osteomyelitis and delayed healing [33,34].

At the molecular level, transcriptomic and metabolomic analyses revealed PINSHIELD's efficacy in attenuating *S.aureus* virulence and adaptability. Transcriptomic analysis showed that PINSHIELD downregulated multiple virulence genes related to biofilm formation and immune evasion, including *cna* and SSL family genes [[35], [36], [37], [38], [39]]. GSEA analysis revealed significant downregulation of bacterial secretion and aminoacyl-tRNA biosynthesis pathways, which are essential for virulence factor secretion and infection persistence [40]. Furthermore, the downregulation of secondary metabolite and nucleotide sugar biosynthesis pathways impaired *S.aureus*' ability to adapt to the infection environment, further confirming PINSHIELD's impact on virulence suppression [41,42]. Metabolomic analysis corroborated these findings by revealing significant inhibition of crucial metabolic pathways involved in fatty acid and unsaturated fatty acid biosynthesis, which are vital for bacterial membrane lipid production and biofilm formation. This metabolic disruption limits *S.aureus*' survival and spread within the host [[43], [44], [45]]. In contrast, the gauze-treated group exhibited upregulation of energy metabolism pathways, such as the TCA cycle, carbon metabolism, and amino acid metabolism, potentially providing *S.aureus* with the metabolic support necessary for biofilm formation and proliferation [[46], [47], [48]]. In the PINSHIELD group, fatty acid degradation was upregulated, suggesting that bacteria may rely on catabolic pathways to maintain essential functions under metabolic suppression, reflecting metabolic reprogramming in response to PINSHIELD's inhibition [49,50]. However, this adaptive response was insufficient to counteract PINSHIELD's broad-spectrum antimicrobial effects. The integrated transcriptome-metabolome analysis revealed that PINSHIELD effectively suppressed key virulence genes, such as SSL family and *cna*, along with associated metabolites like styrene and serotonin, thereby impairing *S.aureus* adhesion and immune evasion capabilities [51]. The upregulation of *nrdD* in response to metabolic stress suggests an adaptive compensatory mechanism, but this limited response was insufficient to counteract PINSHIELD's comprehensive antimicrobial effects [52]. Collectively, these effects are likely mediated by zinc ions released from the ZAPU layer as prior studies have shown that zinc ions, at concentrations exceeding physiological levels, exhibited an antibacterial activity on *E.coli*, *Pseudomonas aeruginosa,* and *S.aureus* [[53], [54], [55], [56]]. The antimicrobial activities are known to be attributed to two mechanisms leading to cell death: the direct destabilization of microbial membranes and increased permeability, as well as the interaction with nucleic acids and deactivation of respiratory enzymes [57]. In parallel, the suppression of aminoacyl-tRNA biosynthesis likely impairs protein translation, further limiting *S.aureus* survival and biofilm formation [58]. Metabolically, zinc ions released from ZAPU may also interfere with bacterial redox balance and carbohydrate metabolism, inducing oxidative stress responses that compromise bacterial viability and virulence [59,60]. Beyond zinc's direct antimicrobial effects, the dual-functional design of PINSHIELD modulates the wound's local microenvironment. By effectively preventing bacterial entry and efficiently managing wound exudates, PINSHIELD indirectly limits nutrient availability and disrupts bacterial quorum-sensing mechanisms, essential for coordinated biofilm formation and infection progression [[61], [62], [63]]. Although precise molecular confirmation of these indirect effects and specific zinc-mediated mechanisms requires further targeted validation studies, our current results robustly support PINSHIELD's ability to disrupt both bacterial virulence and metabolic resilience. Such in-depth mechanistic elucidation represents a critical next step for subsequent studies, strengthening the clinical translation potential of PINSHIELD.

While traditional gauze remains widely used, its limitations in moisture management and antibacterial efficacy necessitate frequent changes and reduced patient compliance [64]. Hydrogels, known for high moisture retention, breathability, and antibacterial properties, have potential in pin-site care but remain underused [65]. Research into antimicrobial coatings for external fixation devices (e.g., antibiotic coatings, calcium hydroxide, or titanium alloy coatings) has shown promising results, but no conclusive evidence has emerged on their ability to prevent infection [66]. PINSHIELD addresses these limitations by integrating multi-layered defense mechanisms, combining physical barriers, antibacterial effects, and exudate control to significantly reduce PSI risk. Its dual-function features offer a considerable advantage in managing pin-site wounds, potentially reducing pin loosening and osteomyelitis, thus improving patient outcomes and reducing hospitalization time and costs [67].

PINSHIELD's versatility suggests broad clinical applications, including external fixation wounds, complex fractures, and other long-term invasive device scenarios. However, practical challenges might arise when translating laboratory findings to diverse clinical settings. For instance, wounds associated with external fixation or bone traction devices vary considerably in exudate volume, bacterial profiles, patient mobility, and underlying health conditions such as diabetes or immunosuppression [68,69]. These variables necessitate tailored dressing strategies to ensure optimal effectiveness of PINSHIELD in different patient populations. Further investigation and clinical trials addressing these complexities will be crucial for the successful clinical translation of PINSHIELD. Moreover, the dual functions of “Top-Sealing” and “Bottom-Draining” provide a novel approach for similar wound care applications, particularly in infection prevention and inflammation management. Future development of PINSHIELD could focus on enhanced material transparency for better wound observation or smart coating materials that dynamically respond to wound changes [[70], [71], [72]]. Integrating Internet of Things (IoT) technology into a smart wound care system could enable real-time monitoring of wound conditions (e.g., temperature, humidity, pH), providing personalized care and timely intervention [73]. Larger-scale clinical trials are needed to evaluate PINSHIELD's efficacy and safety, potentially advancing pin-site wound care and offering more reliable infection prevention strategies [74].

## 4 Materials and methods

### 4.1 Fabrication of PINSHIELD

*Synthesis and Preparation of S*odium Alginate-*Polyurethane Sponge (SAPU), Zinc Alginate-Polyurethane Sponge (ZAPU) and ZAPU-L*.

Polyurethane (PU) sponges (Tianshui Sikon Medical, China) were immersed in sodium alginate (SA) solution (80 mPas, 0.01 g/mL, Qingdao Judah Seaweed Industrial Group Co., Ltd.) for 5 min. Excess sodium alginate was squeezed out, and the sponges were then immersed in zinc chloride (ZnCl_2_) solution (12 mg/mL, Sigma-Aldrich, USAjiduanneir) for 10 s, followed by re-immersion in the SA solution. This procedure was repeated three times, and the sponges were incubated in ZnCl_2_ solution at 37 °C for 2 h before freeze-drying to yield ZAPU.

To prepare SAPU, the PU sponges were immersed in the SA solution (0.01 g/mL) and incubated in an orbital shaker at 37 °C with agitation at 120 rpm for 2 h to facilitate interfacial crosslinking. The samples were then freeze-dried to yield the SAPU.

For the preparation of ZAPU-L, the PU sponges were first immersed in the ZnCl_2_ aqueous solution (12 mg/mL) for 10 min to allow cation adsorption. Afterward, the sponges were immediately transferred to the SA solution (0.01 g/mL) for 2 h. This process resulted in the formation of the blended zinc alginate gelatinized sponge (ZAPU-L).

#### 4.1.1 PINSHIELD assembly

PINSHIELD consists of a stainless-steel clip and silicone shell. The ZAPU sponge was embedded into the silicone shell, and a metal clip was fixed onto the shell. After assembly, the device was weighed. Materials were sourced from Qingdao Ketong Electronics Co., Ltd.

### 4.2 Characterization of PINSHIELD

#### 4.2.1 Mechanical properties

The surface and internal microstructures of different PU materials were examined using scanning electron microscopy (SEM, model JSM-6390LV, Hitachi, Japan). Prior to imaging, the samples were sputter-coated with gold for 160 s and observed at an accelerating voltage of 15.00 kV. The elemental composition of the sponges was analyzed using energy-dispersive X-ray spectroscopy (EDS).

#### 4.2.2 Zinc ion release

Zinc ion concentration was measured using the Elabscience® Zinc (Zn) Colorimetric Assay Kit. Sample extracts were mixed with the reagent, centrifuged, and the supernatant was measured at 545–575 nm using a microplate reader. Zinc ion concentration was calculated using the kit's formula.

#### 4.2.3 Determination of water and serum absorption and retention

Water absorption was evaluated following the YY/T0471.1–2004 method. Dried samples were weighed, immersed in PBS (pH 7.4) for 10 min at 37 °C, and reweighed after 30 s. The water absorption capacity was calculated using the formula: $\text{Swelling}\;\text{rate}\;\left(\%\right)=\frac{m2-m1}{m1}\times 100$

m_1_ = initial mass of the sponge (g).

m_2_ = mass after water absorption (g).

To simulate clinically relevant conditions, the serum absorption capacities of various samples were measured. Additionally, the surface and cross-sectional morphologies of the sponge samples after serum absorption were examined using SEM, following the procedure described previously.

#### 4.2.4 Determination of vapor permeability of water

Water vapor transmission rate (WVTR) was measured following YY/T0471.2–2004. Samples were placed in a centrifuge tube containing distilled water. After incubation at 37 °C for set intervals, the mass change was recorded, and WVTR was calculated using the formula: $\text{WVTR}=\frac{\text{mt}-m0}{S\times t}$

m_0_ = initial mass of the device (g).

m_t_ = mass after time t (g).

*S* = cross-sectional area of the centrifuge tube (mm^2^).

*t* = time (days).

#### 4.2.5 Determination of porosity

The porosity of the samples was determined using the liquid volume displacement method. The sample was weighed, and its volume was calculated based on its dimensions (cylindrical shape, radius r ≈ 1.1 cm, thickness h ≈ 0.5 cm). The initial weight (W_1_) was recorded, and the sample was immersed in anhydrous ethanol for 5 min to absorb the ethanol fully. After removing the sample and gently wiping off excess ethanol, its weight (W_2_) was recorded. The porosity was calculated using the following formula: $\text{Porosity}\left(\%\right)=\frac{w2-w1}{\rho V}\times 100\%$

W_1_ = initial weight (g).

W_2_ = final weight after ethanol immersion (g).

Porosity was calculated from the testing of three parallel samples and expressed as arithmetic mean and standard deviation (M ± SD, n = 3).

### 4.3 *In vitro* antimicrobial performance and safety evaluation

#### 4.3.1 Bacterial barrier effect examination

Cylindrical sponges (10 mm height, 22 mm diameter) were sterilized by UV for 30 min and placed into a clip shell. The assembled devices were sterilized again. The sponges were placed in an acrylic cylinder, with a hyaluronic acid air filtration membrane (HAFM) at the bottom. A bacterial suspension of *S.aureus (*ATCC 29213) and *E*.*coli* (ATCC 8739) (1 × 10^4^ CFU/L, 5 mL) was sprayed into the cylinder. After 1 min, the membrane was removed, placed on LB agar plates, and incubated for 24 h at 37 °C.

#### 4.3.2 Determination of antimicrobial activity

The antimicrobial activity of PU, SAPU, and ZAPU against *S.aureus* and *E.coli* was assessed using a standard colony-forming unit (CFU) counting method. Prior to testing, sponge samples were sterilized under UV light, pre-soaked in either phosphate-buffered saline (PBS) or porcine serum, and then incubated with bacterial suspensions (1 × 10^6^ CFU/mL) at 37 °C for 2 h. Following incubation, the suspensions were serially diluted, and 100 μL of each dilution was plated onto LB agar plates and cultured at 37 °C for 24 h. Bacterial colonies were subsequently counted to evaluate antibacterial efficacy.

#### 4.3.3 Observation of bacteria morphology

The morphological changes of *S. aureus* and *E. coli* following incubation on ZAPU and PU sponge surfaces were examined by SEM. After 4 h of co-culture, the samples were fixed in 2.5 % glutaraldehyde for 2 h, rinsed with deionized water, and sequentially dehydrated in graded ethanol solutions (25 %, 50 %, 75 %, 99 %, and 100 %) for 30 min each. The specimens were then freeze-dried and sputter-coated with gold prior to SEM imaging.

#### 4.3.4 Biocompatibility assay

Cytotoxicity was evaluated using the CCK-8 method. L929 cells were cultured in high-glucose DMEM (Meilun Biotech, China) containing 10 % FBS and 1 % penicillin-streptomycin. The PINSHIELD sponges were soaked in DMEM for 24 h, and the extract was filtered and diluted to various concentrations. L929 cells were seeded in 96-well plates, incubated with the extract, and cell proliferation was assessed after 24 and 72 h using CCK-8. The cell proliferation rate was calculated based on absorbance at 450 nm. The biocompatibility of the ZAPU on L929 cells was subsequently assessed using Calcein-AM/PI staining. L929 cells were inoculated with 1 × 10^4^ cells/dish in confocal petri dishes and ZAPU extract was added and incubated for 24h,72h, after which the supernatant was discarded. Subsequently, Calcein-AM/PI dye was added and incubated for 15 min, and the cell morphology was observed under a fluorescence microscope (TI-S, Nikon, Japan).

#### 4.3.5 Hemolytic assay

Hemolysis was evaluated using anticoagulated whole blood from New Zealand White rabbits. Red blood cells were washed, and 1 mL of red blood cell suspension was prepared with 9 mL PBS. The sample (1 mg) was added to 1 mL diluted blood, and the positive and negative controls were deionized water and PBS, respectively. After incubation at 37 °C for 1 h, the absorbance at 540 nm was measured to calculate the hemolysis rate using the formula: $\text{HR}\left(\%\right)=\frac{ODs-OD\left(-\right)}{OD\left(+\right)-OD\left(-\right)}\times 100\%$

#### 4.3.6 OD_S_ = absorbance of the experimental sample

OD_(−)_ = absorbance of the negative control (PBS).

OD_(+)=_absorbance of the positive control (deionized water).

### 4.4 Porcine pin site infection (PSI) model establishment and treatment

To evaluate the bacterial barrier efficacy of PINSHIELD and its impact on microbial composition, a porcine pin site infection (PSI) model was established. Adult male pigs (approximately 40 kg) were chosen for their similarity to humans in skin structure and wound healing. Anesthesia was induced using 4 % sevoflurane and maintained with 2 % sevoflurane and 1 % oxygen. The hindlimbs were shaved, sterilized with povidone-iodine and 70 % ethanol, and sterile bone pins were inserted into the tibia. After pin insertion, a *S*.*aureus* (ATCC 25923) suspension (1 × 10^8^ CFU/mL) was sprayed onto the pin sites to simulate contamination. Pigs were randomly assigned to either the gauze or PINSHIELD group, and the respective dressing was applied. Animals were monitored daily for clinical signs of infection, and dressing changes were performed every three days. Vital signs, including temperature and weight, were recorded daily. All animal procedures were approved by the Institutional Animal Care and Use Committee (IACUC) of Qingdao University, protocol number QDU-AEC-2024707.

### 4.5 Serum white blood cell, neutrophil, IL-6, and IL-1β concentrations

On day 7, venous blood was collected from the ear vein using sterile 10 mL EDTA-coated vacutainer tubes (Becton Dickinson, USA). The samples were centrifuged at 3000 rpm for 10 min at 4 °C to separate the serum. White blood cell (WBC) and neutrophil counts were determined using an automated hematology analyzer (Mindray BC-5000Vet, Shenzhen, China). Serum IL-6 and IL-1β levels were measured using commercial enzyme-linked immunosorbent assay (ELISA) kits (R&D Systems, Minneapolis, USA), following the manufacturer's instructions. Absorbance was read at 450 nm using a microplate reader (Thermo Fisher Scientific, USA).

### 4.6 CT imaging

After pin removal, surrounding bone and tissue structures were assessed using a dual-energy 128-slice CT scanner (GE Healthcare, USA) with parameters: 200 mA tube current, alternating tube voltage of 140 kVp and 80 kVp, 5 mm slice thickness, 0.625 mm pixel spacing, and 20 cm^2^ DFOV. Scans were averaged over three repetitions. Reconstructed images were analyzed using the AW 4.7 workstation (GE Healthcare, USA). Image storage and retrieval were managed via the PACS system (Picture Archiving and Communication System). The focus was on assessing cortical integrity, density changes, and signs of osteomyelitis or abscess formation.

### 4.7 MRI imaging

MRI scans were performed on a 3.0T MRI system (GE MR750, USA) using multi-channel animal coils. Imaging sequences included T1-weighted (T1WI), T2-weighted (T2WI), and MENSA (Multiple Echoes for Non-Subtracted Acquisition) for enhanced detection of inflammatory areas. Parameters were as follows: TR = 3000 ms, TE = 67.3 ms, FOV = 1 cm × 1 cm, slice thickness = 4 mm, inter-slice spacing = 4.5 mm, matrix size = 96 × 128, SAR = 0.875, and average = 2. Total imaging time was approximately 7 min and 50 s per sample. Images were processed and stored in the PACS system for subsequent analysis.

### 4.8 CT and MRI data analysis and interpretation

CT images were analyzed for cortical disruption, low-density areas indicative of abscesses, and osteolytic changes. MRI scans were evaluated for high-signal intensities on T2-weighted images corresponding to inflammation, and low-signal intensities indicative of gas gangrene. All images were analyzed using PACS-based tools and reviewed by two blinded radiologists with consensus resolution.

### 4.9 Hematoxylin and eosin (HE) staining

Tissue samples from peri-pin tracts were fixed in tissue fixative (Servicebio, G1101-15 ML) for 24 h and dehydrated using a tissue processor (HistoCore PEARL, Leica, Shanghai). Paraffin embedding, sectioning (4 μm), and staining were performed according to standard protocols. Hematoxylin and eosin staining was followed by dehydration and mounting with neutral balsam. Slides were examined using a Nikon ECLIPSE CI microscope (Nikon, Japan) with high-resolution imaging.

### 4.10 IL-1β immunohistochemistry (IHC)

For IL-1β detection, tissue sections were deparaffinized, rehydrated, and subjected to antigen retrieval in citrate buffer (pH 6.0). Endogenous peroxidase was blocked, and sections were incubated with primary IL-1β antibody (Abmart, PK56359M, 1:100) overnight at 4 °C. Secondary antibody (Bioss, bs-0295G-HRP, 1:200) incubation was followed by DAB staining (Beyotime Biotechnology, P0203). Sections were counterstained with hematoxylin and observed under a Nikon ECLIPSE CI microscope (Nikon, Japan).

### 4.11 Bacterial load analysis

Tissue samples were minced and resuspended in 2 mL PBS. The homogenate was diluted (1:10) and spread on solid agar plates. Plates were incubated at 37 °C for 12–16 h, and colony-forming units (CFUs) were counted to determine bacterial load.

### 4.12 Biofilm analysis

Truncated pins were fixed in electron microscope fixative (Sevier, G1102) and rinsed with phosphate buffer (PB, pH 7.4). After osmium acid fixation, samples were dried in a critical point dryer (K850, Quorum) and gold-coated for 240 s under an accelerating voltage of 15.00 kV using an ion sputtering apparatus (MC1000, Hitachi). Biofilm formation was observed using a scanning electron microscope (JSM-6390LV, Nihon Electronics, Japan).

#### 4.12.1 Microbial analysis

DNA Extraction via CTAB Method and Library Construction.

DNA was extracted from peri-pin tract tissue using CTAB lysis buffer with lysozyme. After centrifugation, the supernatant was mixed with phenol-chloroform-isopropanol, followed by chloroform-isoamyl alcohol and isopropanol precipitation. The DNA was washed, air-dried, and dissolved in ddH_2_O. RNase A was added for RNA digestion. PCR amplification of the 16S rRNA V4 region was performed for bacterial diversity assessment. Libraries were constructed using the NEBNext Ultra II FS DNA PCR-free Library Prep Kit (New England Biolabs) and sequenced on the NovaSeq 6000 platform.

#### 4.12.2 Data quality control and denoising

Raw reads were assembled and aligned using FLASH (Version 1.2.11) (Magoc et al., 2011) to generate tags. Low-quality reads were filtered using fastp software (Version 0.23.1) (Bokulich et al., 2012), and denoising was performed in QIIME2 (Version 2022) with the DADA2 plugin to yield high-quality Amplicon Sequence Variants (ASVs).

#### 4.12.3 Bacterial taxonomy annotation and phylogenetic tree construction

Species annotation was performed in QIIME2 using the Silva 138.1 database, and phylogenetic relationships were determined through sequence alignment.

#### 4.12.4 Microbial abundance analysis

Relative abundance of the top 10 taxa was visualized using ggplot2 in R. Heatmaps of the top 35 taxa were generated using the pheatmap package. Venn and petal diagrams were created to visualize group overlap.

#### 4.12.5 Alpha diversity analysis

Alpha diversity indices (observed OTUs, Shannon, Simpson, Chao1, etc.) were calculated in QIIME2, and selected indices were visualized for presentation.

#### 4.12.6 Microbial differential abundance analysis

The Metastats package was employed to assess differential abundance between microbial taxa, with significance set at P < 0.05. Results were visualized using the ComplexHeatmap package in R [75].

#### 4.12.7 *S.aureus* isolation and purification from tissue samples

Tissue (10 mg) was homogenized in sterile saline and centrifuged. The supernatant was plated on Baird-Parker agar to selectively culture *S.aureus*. After 24 h, colonies were purified through two rounds of streaking. DNA was extracted and the 16S rRNA gene amplified for species identification.

### 4.13 Quantitative real-time PCR (qPCR)

To validate the RNA-seq results, qPCR was performed on *S. aureus* isolated from pin-site wounds following PINSHIELD or gauze treatment. qPCR was conducted using qPCR SYBR master mix (Abclonal, CHINA). The following genes were selected for validation: *Cna, Asp2, essA, SSL5* based on RNA-seq data. Gene-specific primers were designed using Primer 5.0 software and their sequences are listed in Supplementary Table S1.

### 4.14 Transcriptomic analysis

#### 4.14.1 RNA isolation and sequencing

Total RNA was extracted using RNA-easy Isolation Reagent (Vazyme Biotech, R701), and RNA integrity was confirmed using a Bioanalyzer. RNA sequencing was performed using the *S*.*aureus* genome reference (GCF_000013425.1) for gene mapping via STAR software [76]. DEGs was analyzed using the edgeR package [77], with genes exhibiting p-value <0.05 and Log2FoldChange >1 considered upregulated and genes exhibiting p-value <0.05 and Log2FoldChange < −1 were downregulated.

#### 4.14.2 Gene function enrichment analysis

Gene functional enrichment analysis was performed using ClusterProfiler [78]. KEGG and GO databases were used to identify enriched pathways and biological functions associated with differentially expressed genes.

### 4.15 Untargeted metabolomics

Metabolomic data were processed in MetaboAnalystR 4.0 for quality control, differential screening, and normalization. Principal Component Analysis (PCA) was conducted to assess sample dispersion. Differential metabolites between gauze and PINSHIELD groups were identified using edgeR, and potential different metabolites were predicted using a Partial Least Squares-Discriminant Analysis (PLS-DA) model with variables of interest identified by VIP >1 and p-value <0.05. KEGG pathway enrichment was performed using the MetaboAnalystR 4.0 pathway module [79].

### 4.16 Transcriptomic and metabolomic integration analysis

Weighted gene co-expression network analysis (WGCNA) was performed using the WGCNA package [80] to calculate the Pearson correlation coefficients between all differential metabolites and genes. Correlations with coefficients >0 were considered positive, while those with coefficients <0 were considered negative. The correlations were ranked by p-value, and visualized for the top-ranked metabolites and genes. Further analysis was performed using the dplyr package to extract subsets of genes related to bacterial secretion and biofilm formation, followed by correlation analysis with all differential metabolites. The top five correlated genes and metabolites were visualized using ggplot2.

KEGG Enrichment of Differential Genes and Metabolites.

Differential genes and metabolites were separately subjected to KEGG pathway enrichment analysis using the ClusterProfiler package. Pathways with p-value <0.05 were selected, and the intersection of KEGG pathways related to both differential genes and metabolites was identified using the dplyr package.

## CRediT authorship contribution statement

**Bing Liang:** Writing – review & editing, Writing – original draft, Funding acquisition, Formal analysis, Conceptualization. **Sha Zhou:** Writing – original draft, Methodology. **Linyuan Xue:** Writing – original draft, Methodology. **Qizun Wang:** Visualization, Project administration, Methodology. **Qianqian Li:** Project administration, Methodology. **Zihan Zheng:** Visualization. **Xinyue Ma:** Visualization, Resources. **Jiyixuan Li:** Investigation. **Li Sun:** Investigation. **Kunyue Xing:** Visualization, Methodology. **Xiaobo Wen:** Visualization, Methodology. **Xiaolin Wu:** Supervision, Methodology, Funding acquisition, Conceptualization. **Miao Zhang:** Writing – review & editing, Methodology. **Dongming Xing:** Writing – review & editing, Project administration, Conceptualization.

## Funding

This study was supported by Shandong Provincial Natural Science Foundation (ZR2023QC117; ZR2022MH218; ZR20220C165), The Affiliated Hospital of Qingdao University Clinical Medicine + X Research Project (QDFY + X2023141; QDFY + X202101054).

## Declaration of competing interest

The authors declare that they have no known competing financial interests or personal relationships that could have appeared to influence the work reported in this paper.

## Data Availability

Data will be made available on request.
