# Supplementary material for: Synchronous bacterial barrier and exudate absorption: A novel dual-function dressing strategy for pin-site infection prevention
Source: Mater Today Bio. 2025 May 8;32:101833. doi: 10.1016/j.mtbio.2025.101833 (PMC12144502; doi:10.1016/j.mtbio.2025.101833)
Supplement: Multimedia component 1 [file mmc1.docx]

**Supplementary Figure**


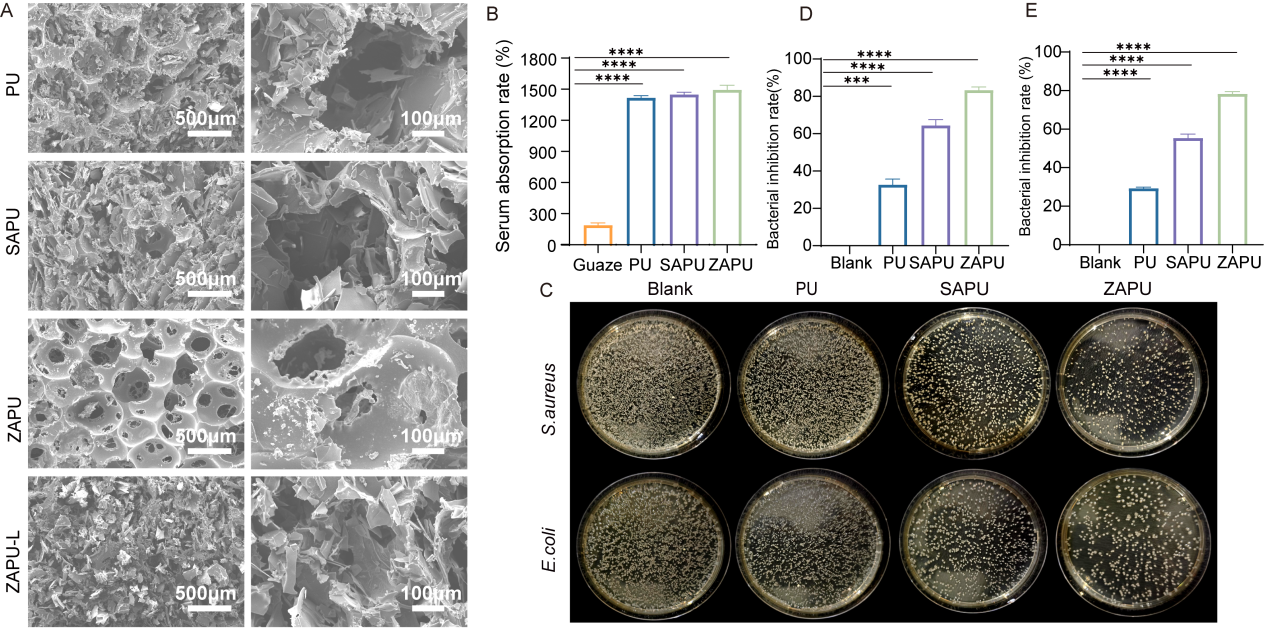


**Fig. S1. Additional characterization of PU, SAPU, ZAPU, and ZAPU-L for exudate handling and antibacterial properties under simulated physiological conditions.** A) SEM images (50x, 200x magnification) of PU, SAPU, ZAPU and ZAPU-L after porcine serum absorption and drying. B) Serum absorption rate of Gauze, PU, SAPU, and ZAPU (****p < 0.0001). C) CFU count of *S.aureus* and *E.coli* on PU, SAPU, and ZAPU. D) Antibacterial rate on *S.aureus* (ZAPU vs Blank, ***p < 0.001, ****p < 0.0001). E) Antibacterial rate on *E.coli* (ZAPU vs Blank, ****p < 0.0001).

**Table S1**

| Gene | Primer sequence |
| --- | --- |
| 16s R | GGTCTGTAACTGACGCTGATGTG |
| 16s F | GTGGACTACCAGGGTATCTAATCCT |
| cna-R | TTTGAGTGCCTTCCCAAACC |
| cna-F | AAAGCGTTGCCTAGTGGAGA |
| essA-R | ACTGCTTCTCTTGATCTTCGC |
| essA-F | GTCTCTTTCACCGCAGCTTT |
| SSL5 R | AGGTGGAAGACACTATCTTGTT |
| SSL5 F | CACCATAAGAATATACAGTGCCGTT |
| asp2 R | AATGCCTGTATCGACGCTTG |
| asp2 F | TGGGTTCATTTGGTGCCTTG |
